# Hyperglycemia Inhibits Complement-Mediated Immunological Control of *S. aureus* in a Rat Model of Peritonitis

**DOI:** 10.1155/2014/762051

**Published:** 2014-12-31

**Authors:** Clifford T. Mauriello, Pamela S. Hair, Reuben D. Rohn, Nicholas S. Rister, Neel K. Krishna, Kenji M. Cunnion

**Affiliations:** ^1^Department of Pediatrics, Eastern Virginia Medical School, 855 West Brambleton Avenue, Norfolk, VA 23501-1980, USA; ^2^Department of Microbiology and Molecular Cell Biology, Eastern Virginia Medical School, 700 West Olney Road, Norfolk, VA 23507, USA; ^3^Children's Specialty Group, 601 Children's Lane, Norfolk, VA 23507, USA; ^4^The Children's Hospital of The King's Daughters, 601 Children's Lane, Norfolk, VA 23507, USA

## Abstract

Hyperglycemia from diabetes is associated with increased risk of infection from *S. aureus* and increased severity of illness. Previous work in our laboratory demonstrated that elevated glucose (>6 mM) dramatically inhibited *S. aureus*-initiated complement-mediated immune effectors. Here we report *in vivo* studies evaluating the extent to which a hyperglycemic environment alters complement-mediated control of *S. aureus* infection in a rat peritonitis model. Rats were treated with streptozocin to induce diabetes or sham-treated and then inoculated i.p. with *S. aureus*. Rats were euthanized and had peritoneal lavage at 2 or 24 hours after infection to evaluate early and late complement-mediated effects. Hyperglycemia decreased the influx of IgG and complement components into the peritoneum in response to *S. aureus* infection and decreased anaphylatoxin generation. Hyperglycemia decreased C4-fragment and C3-fragment opsonization of *S. aureus* recovered in peritoneal fluids, compared with euglycemic or insulin-rescued rats. Hyperglycemic rats showed decreased phagocytosis efficiency compared with euglycemic rats, which correlated inversely with bacterial survival. These results suggest that hyperglycemia inhibited humoral effector recruitment, anaphylatoxin generation, and complement-mediated opsonization of *S. aureus*, suggesting that hyperglycemic inhibition of complement effectors may contribute to the increased risk and severity of *S. aureus* infections in diabetic patients.

## 1. Introduction

*S. aureus* is a major cause of infections in patients with diabetes.* S. aureus* is commonly present in diabetic foot infections [1–4], particularly limb-threatening infections [5–7], as well as more invasive infections like endocarditis [8]. Additionally, hyperglycemia is associated with an increased risk of death from* S. aureus* bacteremia [9]. Thus, diabetes and hyperglycemia appear to increase the risk and severity of infection by* S. aureus* by inhibiting normal host defenses. Currently, these mechanisms are only partially understood.

To date, immunological insufficiency related to hyperglycemia has primarily focused on inhibition of neutrophil responses [10–12]. We have recently shown* in vitro* that hyperglycemic conditions (>6 mM glucose) inhibit complement effectors against* S. aureus* including opsonization and anaphylatoxin generation [13]. Hyperglycemic inhibition of complement-mediated opsonization resulted in decreased phagocytosis efficiency by euglycemic neutrophils, such that neutrophil function was not directly inhibited by excess glucose. Previous investigators had shown that the central component of complement, C3, could be slowly glycated (i.e., 20% glycation over 24 hours) [12]. However, our data demonstrated the effects of elevated glucose on complement activation on the surface of* S. aureus* occurring in minutes. By mass spectrometry we showed that hyperglycemic conditions produced no changes in glycation over one hour, but hyperglycemic conditions did produce changes in the tertiary structure of C3 [13], likely altering its function. These* in vitro* results suggested that the complement system, a major contributor to innate immune host defenses against* S. aureus* [14–16], was significantly inhibited by hyperglycemic conditions in responding to* S. aureus*. Our results were consistent with prior reporting that diabetic patients have a decreased ability to fix complement by IgG [17].

In order to measure the extent to which a hyperglycemic environment altered complement-mediated immune effectors against* S. aureus* infection* in vivo*, we evaluated a diabetic rat peritonitis model. A peritonitis model was chosen as a pertinent clinical model, as well as an excellent model for evaluating complement-mediated effectors [15, 18, 19] by analyzing recovered peritoneal lavage fluid. Previous investigators have evaluated* S. aureus* foot-pad infection in a NOD diabetic mouse model [20], but the mice were C5-deficient limiting the ability to evaluate complement-mediated effects.

## 2. Materials and Methods

### 2.1. Materials

Streptozocin (Sigma Aldrich) was dissolved in saline at 0.5 mg/mL. NPH insulin (Eli Lily) was 10 u/mL.

### 2.2. Bacteria and Growth Conditions

*S. aureus* strain Reynolds was grown in Columbia 2% NaCl broth overnight at 37°C to stationary phase. Colonies were resuspended in saline to 10^8^ CFU/mL.

### 2.3. Animals and Procedures

Animal studies were approved by the EVMS IACUC in accordance with AALAC guidelines. Male Wistar rats (Harlan) were 16 weeks old and 200 grams. A 1 mL suspension of* S. aureus* in saline was injected i.p. Rats were provided acetaminophen in their drinking water (1.6 mg/mL) for analgesia. Rats were monitored for weight, appearance, and behavior, but no animals demonstrated moderate or severe distress during the experiments. Animals were sedated for all procedures with acepromazine-ketamine and euthanized with i.v. FatalPlus. After euthanasia, peritoneal wash was performed percutaneously with 20 mL of ice cold PBS.

Pilot studies were performed with 65 mg/kg streptozocin i.p., which induced stable hyperglycemia after 12 hours and was reversible with 2 units of NPH insulin s.c. Dosing pilot studies were performed with streptozocin-treated rats using 10^7^, 10^8^, or 10^9^ CFU of* S. aureus* i.p., evaluated by peritoneal wash at 6 hours. A dose of 10^8^ CFU demonstrated optimal peritonitis inflammation. The timing of immune-pathogen interactions in streptozocin-treated animals (10^8^ CFU* S. aureus* i.p.) demonstrated optimal evaluation of early events at 2 hours and late events at 24 hours after infection.

**Figure 1:**
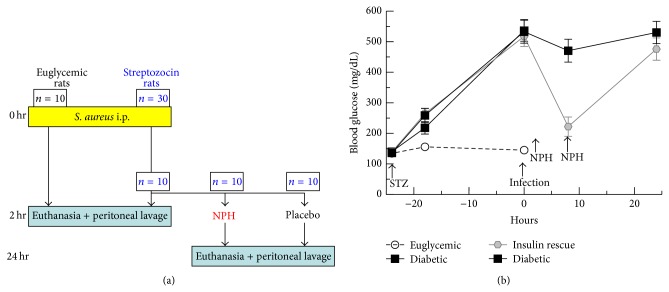
(a) Rat treatment groups and timeline for streptozocin induction, infection, insulin rescue (NPH), and peritoneal lavage. (b) Blood glucose levels for the four groups of rats: euglycemic, diabetic (2 hr), insulin rescue, and diabetic (24 hr). Streptozocin (STZ) was given to all rats except the euglycemic group at −24 hours. Zero hour is time of infection. Insulin (NPH) was given to the insulin rescue group at 2 hours and 8 hours after infection.

A fully powered study was performed with 44 rats (Figure 1(a)). Four rats were untreated and underwent peritoneal wash to measure baseline (0 hour) parameters. Ten (10) rats received sham streptozocin (STZ) injections and 30 rats received STZ 65 mg/mL i.p. 24 hours prior to infection. Stable hyperglycemia was achieved (Figure 1(b)) for all but one STZ-treated rat, which was excluded from subsequent analyses. Thirty-nine (39) rats were inoculated with 10^8^ CFU* S. aureus* i.p. (0 hour). This amount of* S. aureus* is consistent with established* S. aureus* infections [21]. At 2 hours after infection, 19 rats were euthanized and had peritoneal lavage. Two (2) peritoneal wash samples demonstrated particulate matter suggestive of fecal matter and were excluded from subsequent analyses. At 2 hours after infection 10 of the remaining rats were injected with insulin (2 u NPH s.c.), which was repeated (2 u NPH s.c.) at 8 hours after infection. At 24 hours after infection the remaining 20 rats were euthanized and underwent peritoneal wash.

Peritoneal wash samples were placed on ice and then sedimented. Supernatants were recovered, aliquoted, and frozen at −80°C. Pellets were resuspended to their original volume in cold PBS and divided for subsequent analyses. Cytospins (100 *μ*L) were performed to generate multiple slides for each sample. Other aliquots of resuspended pellets were washed with water to lyse neutrophils and then serially diluted for colony count assays or stripped of opsonic C3-fragments and C4-fragments.

### 2.4. Total Leukocyte Counts

Wright stains (Diff-Quik) were performed and 5 random high powered fields were counted for each slide by a blinded observer and averaged. Nearly all leukocytes observed were neutrophils.

### 2.5. Phagocytosis Measurements

Phagocytosis was evaluated by fluorescence microscopy after acridine orange staining of bacteria followed by crystal violet quenching of extracellular bacteria, as previously described [22]. One hundred neutrophils were observed in random high powered fields by a blinded observer and evaluated for the percentage of neutrophils phagocytizing bacteria and the total number of bacteria phagocytized.

### 2.6. Bacterial Counts

Bacterial suspensions were generated from peritoneal wash samples pelleted and resuspended to their original volume in water to lyse neutrophils. The suspensions were then serially diluted and multiple dilutions were plated for colony counting, as previously described [23].

### 2.7. Complement Opsonization of Peritoneal* S. aureus*

*S. aureus* recovered from the peritoneal washes were divided for analysis by flow cytometry, ELISA, or Western blot.* S. aureus* for flow cytometry analysis were incubated with FITC-labeled anti-C3 antibody (MP Biomedicals). Negative controls included (1) unstained peritoneal bacteria and (2) uninjected bacteria incubated with anti-C3 antibody; both yielded minimal fluorescence values below the threshold for detection used for data collection. Fluorescence intensities were measured on a fluorescence activated cell sorter FACSCalibur (BD Biosciences) for 10,000 events. FlowJo software (Tree Star) was used to measure the area under the curve (AUC) for C3-fragments bound to the bacterial surface.

Bound C3-fragments and C4-fragments were stripped from* S. aureus* using methylamine, the most commonly used and accepted methodology, as previously described [24]. The rat C3 ELISA was performed using a rabbit anti-rat C3 antibody for capture (Bethy Lab) and probed with mouse anti-rat C3 antibody (Hycult) followed by goat anti-mouse HRP antibody (Sigma) [25]. Western blot analysis was performed using rabbit anti-rat C3 followed by a goat anti-rabbit HRP antibody. C4 ELISA was performed, as described elsewhere [26]. Quantitation was calculated from standard curves using purified C3 and C4 proteins (CompTech).

### 2.8. Immunoglobulin and Complement Components in Peritoneal Wash Supernatants

Total IgG concentration in peritoneal wash samples was measured by SDS-PAGE and Coomassie staining to minimize backgrounds found with ELISA assay. Peritoneal wash samples and a titration of purified IgG (Gammagard, Baxter) were assayed by SDS-PAGE with Coomassie Brilliant (BioRad) staining. Images were captured digitally and optical densitometry measurements were performed with Quantity One (BioRad). Peritoneal wash samples were measured for total C4 and total C3 by ELISA and assayed by C3 Western blot, as described above.

C3a concentration in peritoneal wash samples was measured by quantitative Western blot to minimize backgrounds found with ELISA. Samples were blotted along with a titration of pure C3a (CompTech) to generate a standard curve. A rabbit anti-human C3a antibody (CompTech) was followed by a goat anti-rabbit HRP antibody (Sigma). Optical densitometry measurements were performed as above. C5a concentration was measured by ELISA (R&D Systems) as per the manufacturer's instructions.

### 2.9. Free DNA in Peritoneal Wash Supernatants

Free DNA in the peritoneal wash supernatants was assayed using Quant-iT PicoGreen (Molecular Probes). Briefly, 1.0 mL of peritoneal wash was combined with 1.0 mL of Quant-iT PicoGreen reagent and fluorescence (excitation 480 nm, emission 520 nm) was measured on a spectrofluorometer (Perkin Elmer). Quantitation was calculated from standard curves using purified DNA.

### 2.10. Myeloperoxidase in Peritoneal Wash Supernatants

Myeloperoxidase activity was measured using TMB Substrate solution [27] (Thermo Scientific). In a 96-well plate, 10 *μ*L of peritoneal wash sample was combined with 100 *μ*L of TMB. The plates were allowed to incubate at room temperature for 30 min and then absorbance values were read at 450 nm.

### 2.11. Statistical Analysis

All data are shown as mean ± standard error of the mean. Comparisons between two groups were made by Student's *t*-test with *P* values ≤ 0.05 considered statistically significant.

## 3. Results

### 3.1. Hyperglycemic Effects on the Influx of Humoral Immune Components to* S. aureus* Peritonitis

**Figure 2:**
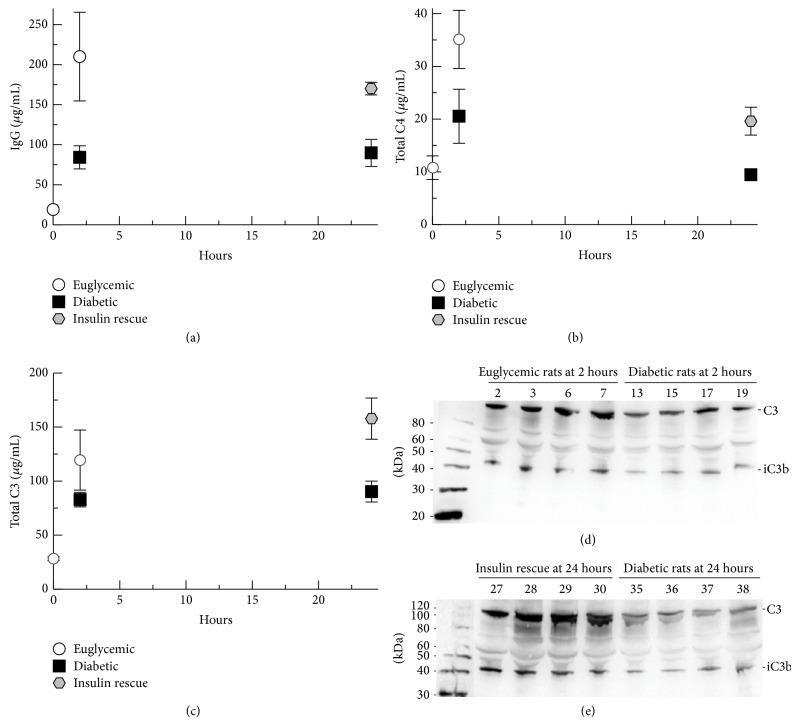
Humoral immune influx into the peritoneum. (a) IgG concentrations in peritoneal wash fluid assayed by SDS-PAGE. (b) Total C4 concentration in peritoneal wash fluid assayed by ELISA. (c) Total C3 concentration in peritoneal wash fluid assayed by ELISA. (d) C3-fragments in peritoneal wash fluid at 2 hours after infection analyzed by Western blot. (e) C3-fragments in peritoneal wash fluid at 24 hours after infection analyzed by Western blot. Graphed data shows means of results for peritoneal samples from the animals in each treatment group. Error bars denote standard error of the means.

In order to evaluate hyperglycemic effects on* S. aureus*-induced influx of humoral immune components critical in the control of* S. aureus* infection, we assayed IgG, C3, and C4 in the peritoneal lavage fluid. Blood glucose levels for the experimental groups are shown in Figure 1(b). All animals survived to the final time point with evidence minimal or no distress. IgG was measured by total protein stained SDS-PAGE (Figure 2(a)), due to high background signal for ELISA. IgG concentration increased from baseline levels to 2 hours after infection. Euglycemic rats showed a 2.5-fold increase in IgG compared with diabetic rats at 2 hours after infection (*P* = 0.03). At 24 hours after infection, insulin-rescued rats showed a 2-fold increase in IgG compared with diabetic rats (*P* = 0.001). These results suggest that hyperglycemia inhibits the influx of IgG to the site of* S. aureus* infection and that insulin treatment of hyperglycemia reverses this effect.

Complement C4, a critical component of classical and lectin complement pathway activation, was assayed by ELISA (Figure 2(b)). At 2 hours after infection, a trend towards increased C4 influx was noted for euglycemic rats compared with diabetic rats (*P* = 0.08). At 24 hours after infection, insulin-rescued rats demonstrated a 2-fold increase in C4 concentration compared with diabetic rats (*P* = 0.02). C3 influx into the peritoneum was assayed by ELISA (Figure 2(c)). At 2 hours after infection, C3 values were not significantly different, but at 24 hours after infection, insulin-rescued rats demonstrated a 1.7-fold increase compared with diabetic rats (*P* = 0.01). In order to confirm C3 ELISA results and evaluate the C3 forms present in the peritoneal fluid, we performed Western blot analyses of the peritoneal fluid. At 2 hours after infection, Western blot analysis suggested increased C3-forms for euglycemic rats compared with diabetic rats and showed that this was predominantly unactivated C3 with some iC3b (Figure 2(d)). Several of these samples were analyzed by Western blot along with purified C3, C3b, and iC3b confirming the identity of C3b and iC3b (data not shown). At 24 hours after infection, Western blot analysis showed increased C3-forms for insulin-rescued rats compared with diabetic rats (Figure 2(e)). Again, the predominant form was unactivated C3 with some iC3b. As a whole, these results suggest that hyperglycemia inhibits the influx of critical complement components to the site of* S. aureus* infection, but insulin rescue can improve the influx of complement components compared with persistent hyperglycemia.

### 3.2. Hyperglycemic Effects on Complement Anaphylatoxin Generation in Response to* S. aureus* Peritonitis

**Figure 3:**
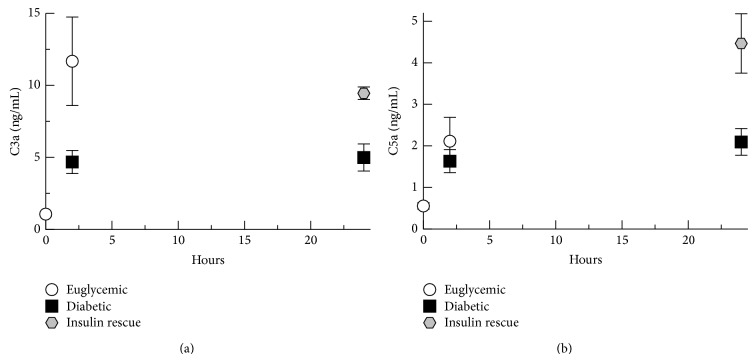
Anaphylatoxin generation in the peritoneum. (a) C3a concentrations in peritoneal wash fluid assayed by quantitative Western blot. (b) C5a concentrations in peritoneal wash fluid assayed by ELISA. Graphed data shows means of results for peritoneal samples from the animals in each treatment group. Error bars denote standard error of the means.

In order to further evaluate hyperglycemic effects on* S. aureus*-initiated complement activation as well as anaphylatoxin generation, we measured C3a and C5a concentrations in the peritoneal lavage samples. Complement anaphylatoxins are direct indicators of complement activation and play vital roles in neutrophil chemotaxis and neutrophil activation, as well as increasing vascular permeability [28, 29]. C3a concentrations were measured using a quantitative Western blot (Figure 3(a)), due to high background levels with ELISA.

Peritoneal C3a levels increased over baseline measurements by 2 hours after infection, at which time C3a concentration in euglycemic rats was 2.5-fold greater than diabetic rats (*P* = 0.03). At 24 hours after infection, C3a concentrations increased to 2-fold which is greater for insulin-rescued rats compared with diabetic rats (*P* = 0.01). At 2 hours after infection C5a levels were not significantly different between the groups (Figure 3(b)), but at 24 hours after infection, C5a concentrations increased to 2-fold greater for insulin-rescued rats compared with diabetic rats (*P* = 0.02). Together, these results show that hyperglycemia was associated with less anaphylatoxin generation in the rat peritoneum in response to* S. aureus* infection. The 24-hour data suggest that insulin rescue and reversal of hyperglycemia significantly increased complement activation and anaphylatoxin generation in response to* S. aureus* infection.

### 3.3. Hyperglycemic Effects on Complement Opsonization of* S. aureus*

**Figure 4:**
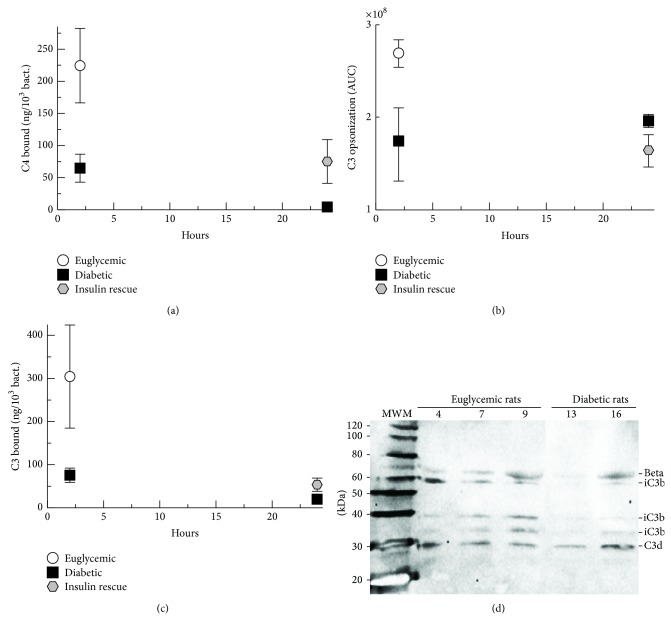
Opsonization of* S. aureus* recovered from the peritoneum. (a) Washed bacteria were stripped of bound C4-fragments and assayed by ELISA. (b) Washed bacteria were analyzed by flow cytometry after incubation with anti-C3 antibody. Area under the curve was measured for each sample (log scale). (c) Washed bacteria were stripped of bound C3-fragments and assayed by ELISA. Graphed data shows means of results for peritoneal samples from the animals in each treatment group. Error bars denote standard error of the means. (d) Total C3-fragment Western blot of stripped bound C3-fragments from representative samples revealing predominantly iC3b and C3d.

In order to evaluate the effects of hyperglycemia on complement opsonization of* S. aureus* in the peritoneum, we recovered the bacteria from the peritoneal wash samples and assayed them by flow cytometry or stripped the bound complement opsonins and assayed them by ELISA and Western blot. Complement-mediated opsonization with C3b and iC3b is critical for efficient phagocytosis of* S. aureus* and survival of bacteremia [22, 23, 30]. Opsonization of* S. aureus* with C4 forms was measured by ELISA and normalized for numbers of bacteria present (Figure 4(a)). C4-fragment opsonization of* S. aureus* was increased by 4-fold at 2 hours for euglycemic rats compared with diabetic rats (*P* = 0.02). C4-opsonization was increased 15-fold for insulin-rescued rats at 24 hours after infection compared with diabetic rats (*P* = 0.05). These results suggest that classical or lectin complement pathway activation on the* S. aureus* surface was greatly decreased in hyperglycemic conditions.

Flow cytometry analysis of efficiency of C3-opsonization of* S. aureus* recovered in the peritoneal wash samples was measured for area under the curve and normalized for 10,000 counts. At 2 hours after infection a 2-fold increase in C3-opsonization was noted for euglycemic rats compared with diabetic rats (*P* = 0.01), but diabetic rats compared with insulin-rescued rats at 24 hours after infection did not show a significant difference (Figure 4(b)). In order to confirm flow cytometry C3-opsonization results, we also performed ELISA measurements of C3-fragments stripped from the* S. aureus* surface and normalized for numbers of bacteria present. Consistent with the flow cytometry data, at 2 hours after infection, a trend towards increased C3-fragment opsonization was noted for euglycemic rats compared with diabetic rats (*P* = 0.06) (Figure 4(c)). At 24 hours after infection, a small but statistically significant increase in C3-fragment opsonization was found for insulin-rescued rats compared with diabetic rats (*P* = 0.05). In order to evaluate the forms of C3 bound to* S. aureus*, we performed C3 Western blot analysis on bacteria recovered at 2 hours after infection. No demonstrable C3b could be identified, but a mixture of opsonic iC3b and nonopsonic C3d was recovered from the surface of* S. aureus* from both euglycemic and diabetic rats (Figure 4(d)). Together these results suggest that hyperglycemia inhibits complement opsonization of* S. aureus* early in infection and that insulin rescue may improve C4-mediated opsonization.

### 3.4. Hyperglycemic Effects on Leukocyte Migration in* S. aureus* Peritonitis

**Figure 5:**
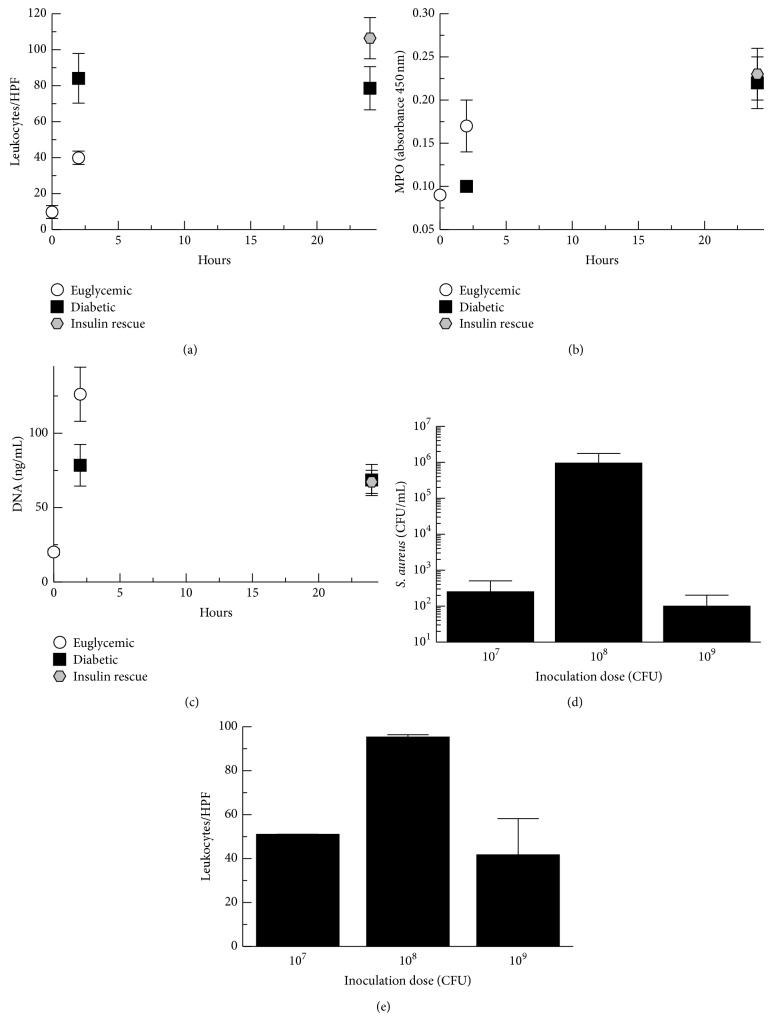
Neutrophil migration into the peritoneum. (a) Leukocytes in the peritoneal fluid wash were quantitated by microscopy of Wright strained cytospin slides. (b) Myeloperoxidase activity (MPO) in the peritoneal fluid wash measured using TMB. (c) Free DNA in the peritoneal fluid wash measured by spectrophotometry using PicoGreen. Graphed data shows means of results for peritoneal samples from the animals in each treatment group. Error bars denote standard error of the means.* S. aureus* inoculation dose ranging experiments with peritoneal wash performed at 24 hours after infection. (d) Leukocyte enumeration of peritoneal wash fluid performed by microscopy of Wright-stained cytospin slides. (e)* S. aureus* recovered from peritoneal wash fluid assayed by colony counting.

Neutrophil migration into the peritoneum in response to complement activation and anaphylatoxin is well established [18, 19, 31]. In order to evaluate hyperglycemic effects on neutrophil migration in response to anaphylatoxin generation, we performed leukocyte counts on peritoneal wash fluids by microscopy of cytospin slides. After 2 hours of infection, nearly all of the leukocytes were neutrophils and both groups showed a considerable increase over baseline value with diabetic rats demonstrating a 2-fold increase in leukocytes compared with euglycemic rats (*P* < 0.01) (Figure 5(a)). The finding of increased neutrophils present for diabetic rats at 2 hours after infection was surprising given the increased levels of C3a present in the peritoneum of euglycemic rats. However, our previous* S. aureus* dose ranging experiments showed that an i.p. dose of 10^9^ CFU yielded fewer recovered bacteria and fewer recovered neutrophils compared with a dose of 10^8^ CFU (Figures 5(d) and 5(e)). Microscopic examination of the slides from rats dosed with 10^9^ CFU demonstrated more cellular debris, suggesting that there may be an increased inflammatory response at 10^9^ CFU resulting in more bacterial killing by neutrophils as well as increased neutrophil death. In order to evaluate whether there could be increased neutrophil death for the euglycemic rats, we measured myeloperoxidase (MPO) activity and free DNA in the peritoneal lavage fluid using TMB and PicoGreen, respectively (Figures 5(b) and 5(c)). At 2 hours after infection, myeloperoxidase activity was increased 1.7-fold for euglycemic rats compared with diabetic rats (*P* = 0.04) and free DNA was increased by 1.6-fold for euglycemic rats compared with diabetic rats (*P* = 0.03). These results suggest that the increased numbers of neutrophils present in the peritoneum of diabetic rats at 2 hours after infection may in part be due to less neutrophil death from activation and degranulation. This is consistent with the expectation that neutrophils in the euglycemic rats will undergo increased phagocytosis and degranulation due to stimulation by C3a compared with diabetic rats.

### 3.5. Hyperglycemic Effects on Neutrophil Phagocytosis and Bacterial Survival in* S. aureus* Peritonitis

**Figure 6:**
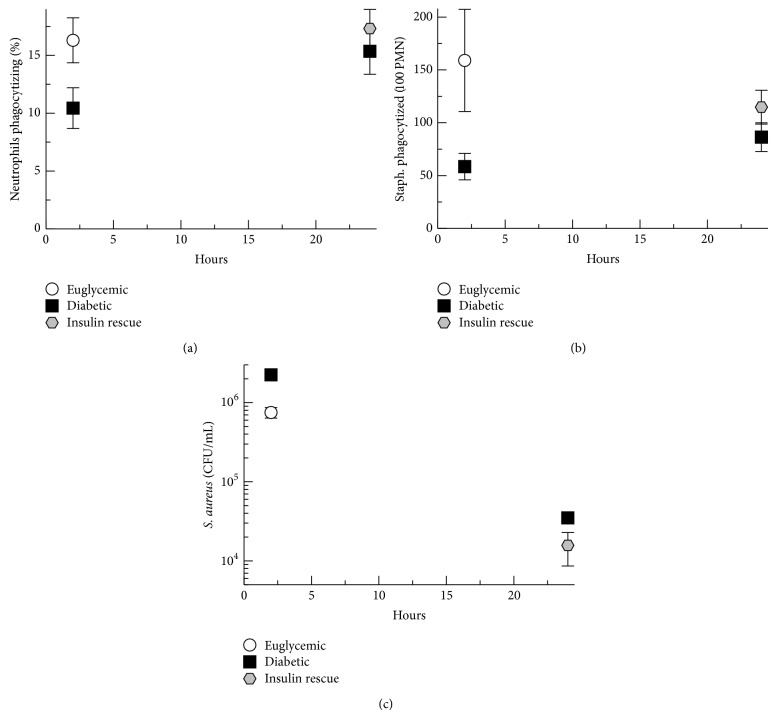
Neutrophil phagocytosis and* S. aureus* survival in the peritoneum. (a) Percent of neutrophils phagocytizing bacteria in peritoneal wash fluid assayed by fluorescence microscopy of acridine orange and crystal violet stained cytospin slides. (b) Bacteria phagocytized per 100 neutrophils in peritoneal wash fluid assayed by fluorescence microscopy of acridine orange and crystal violet stained cytospin slides. (c)* S. aureus* survival assayed by colony counting of peritoneal wash samples. Graphed data shows means of results for peritoneal samples from the animals in each treatment group. Error bars denote standard error of the means.

In order to evaluate the downstream effect of hyperglycemic inhibition of complement effectors against* S. aureus*, we measured phagocytosis efficiency and bacterial survival. We have previously shown* in vitro* that hyperglycemic inhibition of complement opsonization of* S. aureus* inhibits phagocytosis by euglycemic neutrophils [13]. To evaluate the* in vivo* effects of hyperglycemia on neutrophil function in this rat model, neutrophil phagocytosis efficiency was assayed by fluorescence microscopy of acridine orange and crystal violet stained cytospin slides of peritoneal wash samples. At 2 hours after infection, euglycemic rats demonstrated a 1.6-fold increase in neutrophils phagocytosis of bacteria (Figure 6(a)) compared with diabetic rats (*P* = 0.02). At 24 hours after infection there was no significant difference between groups. At 2 hours after infection, the number of bacteria phagocytized by 100 neutrophils (Figure 6(b)) was increased 3-fold in euglycemic rats compared with diabetic rats (*P* = 0.02). There was a nonsignificant trend towards increased numbers of bacteria phagocytized at 24 hours after infection in insulin-rescued rats (*P* = 0.1).

*S. aureus* survival in the rat peritoneum was assessed by colony counting of peritoneal wash samples. At 2 hours after infection,* S. aureus* survival was 3-fold higher in diabetic rats compared with hyperglycemic rats (*P* = 0.03) (Figure 6(c)). At 24 hours after infection there was no statistically significant difference in* S. aureus* survival between diabetic and insulin-rescued rats. Taken together, these results suggest that hyperglycemic inhibition of complement effectors in* S. aureus* peritonitis may contribute to decreased phagocytosis efficiency and increased bacterial survival, at least early in infection.

## 4. Discussion

These experiments were designed to evaluate the effects of hyperglycemia on complement effectors against* S. aureus* in a rat model of peritoneal infection. Hyperglycemia adversely affected the early (i.e., 2-hour) influx of critical humoral immune components to the site of* S. aureus* infection. A likely explanation for this effect is hyperglycemia inhibiting* S. aureus* activation of complement components already present in the peritoneum at the time of inoculation. Previous investigators have shown that complement activation in the peritoneum greatly increases vascular permeability and leakage of plasma proteins via anaphylatoxin [32]. Our data show there are low concentrations of IgG, C4, and C3 present in peritoneal fluid in the absence of infection. Hyperglycemia inhibition of* S. aureus*-initiated complement activation at the time of inoculation, as suggested by our* in vitro* data [13], would decrease the amount of anaphylatoxins generated by the complement components already present in the peritoneal fluid. Uninhibited anaphylatoxin generation, as would be expected for the euglycemic animals, should increase histamine release and increase vascular permeability [33] facilitating the extravascular transit of plasma components. Such a mechanism would be consistent with our C3a findings. Evaluation of these relatively short-lived anaphylatoxins minutes after infection could potentially be more revealing. Reversal of hyperglycemia by administering insulin improved the influx of humoral immune components to the site of infection at 24 hours after infection compared with animals that did not receive insulin. C3a and C5a generations were both increased at 24 hours after infection. These results suggest that reversing hyperglycemic conditions may enhance anaphylatoxin generation and increase humoral immune component influx to the site of infection. Thus, these results suggest that hyperglycemia inhibits early humoral responses to* S. aureus* infection and that these effects can be reversed, at least partially, with insulin therapy.

Opsonization of* S. aureus* in euglycemic rats was increased for both C4-fragments and C3-fragments at 2 hours after infection compared with diabetic rats. This correlated with the increased C3a levels for euglycemic animals indicating increased complement activation. These findings demonstrate that a hyperglycemic environment inhibits* S. aureus*-initiated complement activation and opsonization of* S. aureus*, consistent with our prior* in vitro* findings [13]. Decreased C4-fragment opsonization in hyperglycemic conditions suggests that the classical pathway, or lectin pathway, or both, may have been inhibited. Prior studies have suggested that high glucose conditions will inhibit MBL binding to mannan [34]. It is also possible that high glucose environments may have a direct effect on the C4 molecule, as we demonstrated [13] for the evolutionarily related C3 molecule [33]. Alternatively, excess glucose may alter the surface of microbial pathogens inhibiting complement effectors, as has been shown for* Candida albicans* [35, 36].

Phagocytosis of* S. aureus* by neutrophils was increased at 2 hours after infection for euglycemic rats compared with diabetic rats. This correlated inversely with* S. aureus* survival where diabetic rats showed increased numbers of live bacteria at 2 hours after infection compared with euglycemic rats. We have previously shown that hyperglycemic conditions inhibit complement-mediated opsonization of* S. aureus* resulting in decreased phagocytosis by euglycemic neutrophils, where neutrophil function was not affected by excess glucose [13]. In these* in vivo* experiments, hyperglycemia may have blunted neutrophil function to some extent. However, our prior* in vitro* findings and present demonstration of decreased complement opsonization of* S. aureus in vivo* suggest that hyperglycemic inhibition of complement responses to* S. aureus* likely contributed to decreased phagocytosis and increased bacterial survival for diabetic rats.

**Figure 7:**
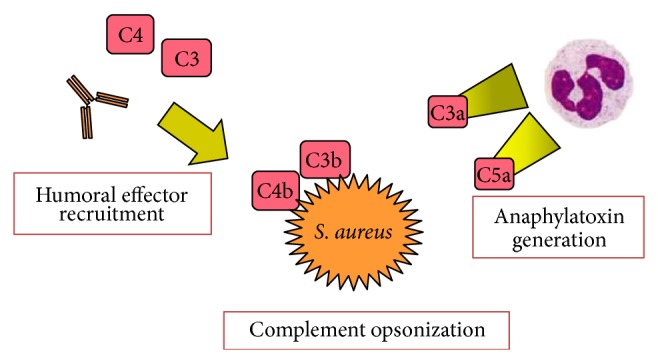
Summary of hyperglycemic effects on humoral immune responses to* S. aureus* peritonitis. Hyperglycemia inhibited antibody and complement effector recruitment to the site of infection. Hyperglycemia inhibited complement opsonization of* S. aureus* and complement anaphylatoxin generation.

In summary, these rat experiments show that hyperglycemia inhibited humoral effector recruitment, complement-mediated opsonization of* S. aureus*, and complement anaphylatoxin generation (Figure 7). Treatment with insulin after infection improved some of these complement effectors compared with persistent hyperglycemia.
